# Supplementary material for: Simplifying drone-based aboveground carbon density measurements to support community forestry
Source: PLoS One. 2025 Apr 29;20(4):e0322099. doi: 10.1371/journal.pone.0322099 (PMC12040082; doi:10.1371/journal.pone.0322099)
Supplement: S1 Text — (DOCX) [file pone.0322099.s001.docx]

**S1 Text. Processing parameters for tree crown location estimates generated using PyCrown.**

Table A shows the different processing parameters used for the five PyCrown-derived tree crown location estimates. We resampled the canopy height model (CHM) from 0.1 m to either 0.25 m or 0.5 m resolution to reduce processing time and memory requirements. The width, in pixels, of the moving window we used to detect local maxima in the CHM varied from 5 to 10. We set the minimum tree height (the threshold below which a pixel cannot be a local maxima) to be lower than the minimum tree height from the field measurements (3.7 m) to ensure that we captured all trees measured by the field team, but high enough to reduce the inclusion of bushes and other vegetation.

**Table A. PyCrown parameters.**

| Estimate number | | Resolution (m) | Window size (pixels) | | Minimum tree height (m) |
| --- | --- | --- | --- | --- | --- |
| 1 | 0.5 | | | 5 | 2 |
| 2 | 0.25 | | | 5 | 3 |
| 3 | 0.25 | | | 7 | 3 |
| 4 | 0.25 | | | 8 | 3 |
| 5 | 0.25 | | | 10 | 3 |
