# Supplementary material for: Simplifying drone-based aboveground carbon density measurements to support community forestry
Source: PLoS One. 2025 Apr 29;20(4):e0322099. doi: 10.1371/journal.pone.0322099 (PMC12040082; doi:10.1371/journal.pone.0322099)
Supplement: S1 Table — AGB in kg; DBH, diameter at breast height in cm; H, tree height in m; WD, wood density in g cm3; W, weight in kg. Forest types and underlying sample data ranges are given where available. (DOCX) [file pone.0322099.s002.docx]

**S1 Table. Selected allometric equations used to generate aboveground biomass (AGB) distributions from field-derived measurements.**

| Equation | Model type | Forest type | Sample data range | AGB equation | Reference |
| --- | --- | --- | --- | --- | --- |
| 1 | DBH | Moist forests | *n* = 170;  DBH range: 5-148 cm | $AGB=\exp(-2.134+2.530\times\ln\left( DBH \right))$ | [1] |
| 2 |  | Moist tropical forests | *n* = 266;  DBH range: 5-148 cm | $AGB=\exp(-2.289+2.649\times\ln\left( DBH \right)-0.021 \times{(\ln\left( DBH \right))}^{2})$ | [2] |
| 3 |  | Secondary tropical forest, Kalimantan | WD range: 0.29-0.47 g cm^-3^ | $AGB=\exp(-2.51+2.44\times\ln\left( DBH \right))$ | [3] |
| 4 |  | Peat swamp forest, Kalimantan | DBH range: 2-35 cm | $AGB=0.107\times{DBH}^{2.486}$ | Jaya et al., 2007 in  [4] |
| 5 |  | Logged over tropical forest, Sarawak | *n* = 30; 27 spp;  DBH range: 1-44.1 cm;  H range: 1.5-40 m | $AGB=0.1525\times{DBH}^{2.34}$ | [5] |
| 6 |  | Logged over peat swamp forest, Sarawak | *n* = 41;  DBH range: 5.2-110.8 cm;  H range: 7.1-49.4 m | $AGB=\exp(-1.4927+2.2250\times\ln\left( DBH \right))$  Correction factor = 1.0581 | [6] |
| 7 |  | Logged over peat swamp forest, Sarawak | *n* = 41;  DBH range: 5.2-110.8 cm;  H range: 7.1-49.4 m | $AGB=\exp\left( -3.5339+3.7146\times\ln\left( DBH \right)-0.2529\times\left( \ln\left( DBH \right) \right)^{2} \right)$  Correction factor = 1.0459 | [6] |
| 8 |  | Primary rainforest, Kalimantan | WD range: 0.36-0.81 g cm^-3^ | $AGB=\exp(-2.30+2.62\times\ln\left( DBH \right))$ | [7] |
| 9 | DBH.H | Moist forests | *n* = 168 | $AGB=\exp(-3.1141+0.9719\times\ln\left( {DBH}^{2}\times H \right))$ | [8] |
| 10 |  | Peat swamp forest, Kalimantan | *n* = 30 | $W_{stem}=0.014\times{(DBH\times H)}^{1.569}$ | [9] |
|  |  |  |  | $W_{branch}=0.0036\times{(DBH\times H)}^{1.513}$ |  |
|  |  |  |  | $W_{twig}=0.0076\times{(DBH\times H)}^{1.1937}$ |  |
|  |  |  |  | $W_{leaf}=0.028\times{(DBH\times H)}^{0.8803}$ |  |
|  |  |  |  | $AGB=W_{stem}+W_{branch}+W_{twig}+W_{leaf}$ |  |
| 11 |  | Logged over tropical forest, Sarawak | *n* = 30; 27 spp;  DBH range: 1-44.1 cm;  H range: 1.5-40 m | $AGB=0.1083\times{({DBH}^{2}\times H)}^{0.80}$ | [5] |
| 12 |  | Peat swamp forest, Indonesia | *n* = 148;  DBH range: 2-167 cm;  H range: 2.8-49.5 m | $AGB=0.081\times{DBH}^{2.049}\times H^{0.672}$ | [10] |
| 13 |  | Hardwood species, not dipterocarps, peat swamp forest, Indonesia | *n* = 62;  DBH range: 2-167 cm;  H range: 2.8-36.2 m;  hardwood WD > 0.543 g cm^-3^ | $AGB=0.057\times{DBH}^{1.815}\times H^{1.085}$ | [10] |
| 14 |  | Logged over peat swamp forest, Sarawak | *n* = 41;  DBH range: 5.2-110.8 cm;  H range: 7.1-49.4 m | $AGB=\exp\left( -3.8903+3.1700\times\ln\left( DBH \right)+0.5108\times\ln\left( H \right)-0.2013\times\left( \ln\left( DBH \right) \right)^{2} \right)$  Correction factor = 1.0444 | [6] |
| 15 |  | Peat swamp forest, Riau | *n* = 51; 11 spp;  DBH range: 5.2-62.7 cm;  H range: 6.4-39.4 m | $AGB=\exp(-3.398+0.995\times\ln\left( {DBH}^{2}\times H \right))$ | [11] |
| 16 |  | Peat swamp forest, Riau | *n* = 51; 11 spp;  DBH range: 5.2-62.7 cm;  H range: 6.4-39.4 m | $AGB=\exp(-3.580+1.827\times\ln\left( DBH \right)+1.229 \times\ln\left( H \right))$ | [11] |
| 17 | DBH.WD | Moist tropical forests | *n* = 1504;  max DBH = 156 cm; | $AGB=WD\times\exp(-1.499+2.148\times\ln\left( DBH \right)+0.207\times{(\ln\left( DBH \right))}^{2}-0.0281\times\left( \ln\left( DBH \right) \right)^{3})$ | [12] |
| 18 | DBH.H.WD | Moist forests | *n* = 94 | $AGB=\exp(-2.4090+0.9522\times\ln\left( {DBH}^{2}\times H\times WD \right))$ | [8] |
| 19 |  | Moist tropical forests | *n* = 1265;  max DBH = 156 cm | $AGB=0.0509\times{DBH}^{2}\times H\times WD$ | [12] |
| 20 |  | Pantropical forests | *n* = 4004;  DBH range: 5-156cm | $AGB=0.673\times{({DBH}^{2}\times H\times WD)}^{0.976}$ | [13] |
| 21 |  | Peat swamp forest, Kalimantan | *n* = 30 | $W_{stem}=0.037\times{(DBH\times H\times WD)}^{1.523}$ | [9] |
|  |  |  |  | $W_{branch}=0.008\times{(DBH\times H\times WD)}^{1.486}$ |  |
|  |  |  |  | $W_{twig}=0.0146\times{(DBH\times H\times WD)}^{1.174}$ |  |
|  |  |  |  | $W_{leaf}=0.0466\times{(DBH\times H\times WD)}^{0.8503}$ |  |
|  |  |  |  | $AGB=W_{stem}+W_{branch}+W_{twig}+W_{leaf}$ |  |
| 22 |  | Peat swamp forest, Indonesia | *n* = 148;  DBH range: 2-167 cm;  H range: 2.8-49.5 m | $AGB=0.15\times{DBH}^{2.095}\times H^{0.552}\times{WD}^{0.664}$ | [10] |
| 23 |  | Hardwood species, not dipterocarps, peat swamp forest, Indonesia | *n* = 62;  DBH range: 2-167 cm;  H range: 2.8-36.2 m;  hardwood WD > 0.543 g cm^-3^ | $AGB=0.077\times{DBH}^{1.871}\times H^{1.008}\times{WD}^{0.669}$ | [10] |
| 24 |  | Logged over peat swamp forest, Sarawak | *n* = 41;  DBH range: 5.2-110.8 cm;  H range: 7.1-49.4 m | $AGB=\exp(-2.3785+1.7078\times ln \left( DBH \right)+0.9704\times\ln\left( H \right)+0.6389\times\ln\left( WD \right))$  Correction factor = 1.0487 | [6] |
| 25 |  | Logged over peat swamp forest, Sarawak | *n* = 41;  DBH range: 5.2-110.8 cm;  H range: 7.1-49.4 m | $AGB=\exp(-3.4804+3.0581\times\ln\left( DBH \right)+0.6242\times\ln\left( H \right)+0.6405\times\ln\left( WD \right)-0.2015\times\left( \ln\left( DBH \right) \right)^{2})$  Correction factor = 1.0425 | [6] |
| 26 |  | Peat swamp forest, Riau | *n* = 51; 11 spp;  DBH range: 5.2-62.7 cm;  H range: 6.4-39.4 m | $AGB=\exp(-2.965+0.990\times\ln\left( {DBH}^{2}\times H\times WD \right))$ | [11] |
| 27 |  | Peat swamp forest, Riau | *n* = 51; 11 spp;  DBH range: 5.2-62.7 cm;  H range: 6.4-39.4 m | $AGB=\exp(-3.126+2.011\times\ln\left( DBH \right)+0.966 \times\ln\left( H \right)+0.641\times\ln(WD))$ | [11] |

AGB in kg; DBH, diameter at breast height in cm; H, tree height in m; WD, wood density in g cm^3^; W, weight in kg. Forest types and underlying sample data ranges are given where available.

**References**

1. Brown S. Estimating biomass and biomass change of tropical forests: A primer. Rome; 1997.

2. Brown S, Casarim FM, Grimland SK, Pearson T. Carbon Impacts from Selective Logging of Forests in Berau, East Kalimantan, Indonesia. Arlington; 2011.

3. Hashimoto T, Tange T, Masumori M, Yagi H, Sasaki S, Kojima K. Allometric equations for pioneer tree species and estimation of the aboveground biomass of a tropical secondary forest in East Kalimantan. Tropics. 2004;14: 123–130. doi:10.3759/tropics.14.123

4. Krisnawati H, Adinugroho WC, Imanuddin R. Allometric Models for Estimating Tree Biomass at Various Forest Ecosystem Types in Indonesia. English. Bogor: Research and Development Center for Conservation and Rehabilitation; 2012.

5. Kenzo T, Ichie T, Hattori D, Itioka T, Handa C, Ohkubo T, et al. Development of allometric relationships for accurate estimation of above- and below-ground biomass in tropical secondary forests in Sarawak, Malaysia. J Trop Ecol. 2009;25: 371–386. doi:10.1017/S0266467409006129

6. Monda Y, Kiyono Y, Melling L, Damian C, Chaddy A. Allometric equations considering the influence of hollow trees: A case study for tropical peat swamp forest in Sarawak. Tropics. 2015;24: 11–22. doi:10.3759/tropics.24.11

7. Yamakura T, Hagihara A, Sukardjo S, Ogawa H. ABoveground Biomass of Tropical Rain Forest Stands in Indonesian Borneo. Vegetatio. 1986;68: 71–82. doi:10.2307/20037339

8. Brown S, Gillespie AJR, Lugo AE. Biomass Estimation Methods for Tropical Forests with Applications to Forest Inventory Data. For Sci. 1989;35: 881–902. doi:10.1093/forestscience/35.4.881

9. Dharmawan IWS, Darusman T, Naito R, Arifanti VB, Lugina M, Hartoyo ME. ITTO Project Technical Report PD 73/89 (F, M, I) Phase II: Development and testing of a carbon MRV methodology and monitoring plan. Bogor; 2012.

10. Manuri S, Brack C, Nugroho NP, Hergoualc’h K, Novita N, Dotzauer H, et al. Tree biomass equations for tropical peat swamp forest ecosystems in Indonesia. For Ecol Manage. 2014;334: 241–253. doi:10.1016/j.foreco.2014.08.031

11. Nugroho NP. Developing Site-specific Allometric Equations for Above-ground Biomass Estimation in Peat Swamp Forests of Rokan Hilir District, Riau Province, Indonesia. Indones J For Res. 2014;1. doi:10.20886/ijfr.2014.1.1.47-65

12. Chave J, Andalo C, Brown S, Cairns MA, Chambers JQ, Eamus D, et al. Tree allometry and improved estimation of carbon stocks and balance in tropical forests. Oecologia. 2005;145: 87–99. doi:10.1007/s00442-005-0100-x

13. Chave J, Réjou-Méchain M, Búrquez A, Chidumayo E, Colgan MS, Delitti WBC, et al. Improved allometric models to estimate the aboveground biomass of tropical trees. Glob Chang Biol. 2014;20: 3177–3190. doi:10.1111/gcb.12629
