# Supplementary material for: Simplifying drone-based aboveground carbon density measurements to support community forestry
Source: PLoS One. 2025 Apr 29;20(4):e0322099. doi: 10.1371/journal.pone.0322099 (PMC12040082; doi:10.1371/journal.pone.0322099)
Supplement: Attachment — Submitted filename: PONE-D-24-34507_reviewer.docx [file pone.0322099.s003.docx]

**PLOS ONE**

Manuscript	[Click here to](https://www2.cloud.editorialmanager.com/pone/download.aspx?id=37217384&guid=d1d12697-456c-4cb0-9208-d189679c8786&scheme=1)

[access/download;Manuscript;Manuscript_BN_24.docx](https://www2.cloud.editorialmanager.com/pone/download.aspx?id=37217384&guid=d1d12697-456c-4cb0-9208-d189679c8786&scheme=1)

1	Simplifying drone-based aboveground carbon density measurements to support community forestry 2

3	Ben Newport^1^*, Tristram C. Hales^2^, Joanna House^1,3^, Benoit Goossens^4,5,6^, Amaziasizamoria Jumail^4,5^ 4

5 ^1^ School of Geographical Sciences, University of Bristol, Bristol, UK
6 ^2^ School of Earth and Environmental Sciences, Cardiff University, Cardiff, UK
7 ^3^ Cabot institute, University of Bristol, Bristol, UK
8 ^4^ Organisms and Environment Division, School of Biosciences, Cardiff University, Cardiff, UK
9 ^5^ Danau Girang Field Centre, c/o Sabah Wildlife Department, Kota Kinabalu, Malaysia
10 ^6^ Sabah Wildlife Department, Kota Kinabalu, Malaysia 11
12 * Corresponding author
13 email: [ben.newport@bristol.ac.uk](mailto:ben.newport@bristol.ac.uk)

# 14 Abstract

15 Community-based Forest restoration has the potential to sequester large amounts of
16 atmospheric carbon, avoid forest degradation, and support sustainable development. However, if
17 partnered with international funders, such projects often require robust and transparent
18 aboveground carbon measurements to secure payments, and current monitoring approaches are not
19 necessarily appropriate due to costs, scale, and complexity. The use of consumer-grade drones in
20 combination with opensource structure-from-motion photogrammetry may provide a solution. In
21 this study, we tested the suitability of a simplified drone-based method for measuring aboveground
22 carbon density in heavily degraded tropical forests at a 2 ha restoration site in Sabah, Malaysia,
23 comparing our results against established field-based methods. We used structure-from-motion
24 photogrammetry to generate canopy height models from drone imagery, and applied multiple pre-
25 published plot-aggregate allometric equations to examine the importance of utilizing regionally
26 calibrated allometric equations. Our results suggest that this simplified method can produce
27 aboveground carbon density measurements of a similar magnitude to field-based methods, quickly
28 and only with a single input metric, albeit with greater levels of uncertainty. Our findings also
29 highlight the importance of selecting regionally calibrated allometric equations for this approach. At
30 scales between 1 and 100 ha, drone-based methods provide an appealing option for data acquisition
31 and carbon measurement, balancing trade-offs between accuracy, simplicity, and cost effectiveness
32 and coinciding well with the needs of community-scale aboveground carbon measurement. Of
33 importance, we also discuss considerations relating to the accessibility of this method for community
34 use, beyond purchasing a drone, that must not be overlooked. Nevertheless, the method presented
35 here lays the foundations for a simple workflow for measuring aboveground carbon density at a
36 community scale that can be refined in future studies.

# 37 Introduction

38 Small-scale, community-based forest restoration can
39 sequester large amounts of atmospheric carbon, reduce emissions from deforestation and
40 degradation, and support sustainable development [1–5]. Community-scale projects typically cover
41 tens of hectares or less and are implemented by stakeholders, including community groups,
42 villages, and NGOs. Numbering in the tens of thousands globally, such projects are important for two
43 key reasons. Firstly, they involve indigenous and rural communities in forest
44 management, a key factor in enhancing both the ecological and social
45 outcomes of restoration activities [6–8]. Secondly, forests restored in this manner are more likely to
46 persist into the long-term (decades to centuries) than large-scale tree planting projects developed
47 without community support [9–12]. Many community-scale projects partner with funders from
48 developed nations who provide financial compensation to support climate and sustainability-
49 oriented goals such as carbon offsetting. These financial mechanisms require projects to provide
50 robust biomass measurements to verify baseline carbon values at restoration sites [13,14]. However,
51 current established methods for measuring aboveground carbon density (ACD, often reported in Mg
52 C ha^-1^) are not necessarily appropriate for use at the community scale, are time consuming, and
53 require specialist training.
54 Several methods are currently used to quantify ACD in forest stands, including both field-
55 based and remote sensing surveys of tree metrics. The increasing availability and accessibility of
56 remote sensing data make this an important tool for forest restoration. Remotely-sensed variables
57 are used to calculate ACD via a series of empirical allometric equations supported by statistical
58 analysis based on ACD values from permanent field plots [15–18]. The benefit of using remote
59 sensing is that it can be employed over large scales and in remote areas, and is often cheaper and
60 more feasible than extensive ground surveys. While such an approach has been employed
61 extensively by academics and commercial foresters, it presents challenges for use at a community
62 scale. The cost of procuring high-resolution (<3 m) remote sensing imagery suitable for community-
63 scale carbon quantification can be prohibitively expensive for community-scale actors. Freely
64 available datasets (e.g., Landsat, GEDI) may have too coarse a resolution for meaningful or timely
65 analysis, with low resample rates exacerbated by persistent cloud cover in the tropics [19,20].
66 Lightweight, low-cost, consumer-grade drones (also known as unmanned [sic] aerial vehicles
67 (UAVs) [although see 21]) offer a potential solution to these data acquisition issues. Consumer-grade
68 drones are relatively cheap (to purchase and to operate) compared to other data collection methods;
69 they can be piloted with minimal training and a smartphone; they have high spatial and temporal
70 resolution; and they grant autonomy over data collection, an important step in empowering and
71 engaging local people in conservation initiatives [22,23]. In addition, the optical imagery that drones
72 generate can be combined with structure-from-motion (SfM) photogrammetry – which produces 3D
73 point clouds from sets of overlapping 2D images [24] – to calculate canopy height and, subsequently,
74 carbon values in a similar manner to other remote sensing approaches [25–27]. Drone-based SfM is
75 a good potential fit for community-scale ACD measurement as it does not require information on
76 camera location and orientation, enabling the use of inexpensive platforms and sensors [24,28,29].
77 However, remote sensing-based ACD quantification methods often involve generating novel
78 allometric equations [18,30] which may be challenging for community-scale projects with low levels
79 of external support. The use of pre-published allometric equations offers an alternative option and
80 they are frequently used in field-based individual tree crown (ITC) measurements, either out of
81 convenience or necessity [31]. Yet, to date, there have been few studies investigating the accuracy
82 and uncertainties surrounding the use of pre-published plot-aggregate allometric equations with
83 drone-derived SfM data for small-scale ACD measurements.
84 In this study we assess the suitability of a simplified method for measuring ACD within the
85 context of community-scale forest restoration, using a consumer-grade drone and opensource SfM
86 software. We compare our results against field-based measurements of ACD to examine their biases
87 and uncertainties. We use a restoration site in Malaysian Borneo as a case study site, representing a
88 real-world restoration project where this method would be applicable. In this context, this study not only fills a gap in the literature regarding drone-based ACD measurements at the community scale, but also contributes to practical insights for restoration practitioners in tropical forest restoration.

90	[32,33].

# 91 Materials and methods

## 92 Study region

93 We calculated different drone-derived carbon metrics within a 2 ha forest restoration plot in the Pin
94 Supu Forest Reserve (4,696 ha), part of the Lower Kinabatangan Wildlife Sanctuary, Sabah, Malaysia
95 (5°25′15″ N 117°58′05″ E) (Fig 1). The restoration site, known as Kaboi Lake, is managed by the
96 charity Regrow Borneo ([www.regrowborneo.org](http://www.regrowborneo.org/)), the Danau Girang Field Centre (DGFC), and the
97 Community Ecotourism Co-operative of the Batuh Puteh Community (KOPEL). Located within the
98 Kinabatangan floodplain, the site is a seasonally flooded freshwater swamp forest. The site has an
99 average annual rainfall of 2700 mm with an average temperature of 25.7 °C [34], and total relief
100 across the site is <1 m. Kaboi Lake lacks any dipterocarps (Dipterocarpaceae family), a numerically
101 dominant and carbon-dense tree family in Borneo [35,36], due to selectively logged in the
102 1980s [37]. Kaboi Lake and the surrounding forest were gazetted by the Sabah Forestry Department
103 (SFD) in 1984 and has since been left to regenerate naturally [38]. In 2020, KOPEL team members cleared the site for replanting, removing elephant grass (*Pennisetum purpureum*), climbing
104 bamboo (*Dinochloa spp.*) and various vines to make way for flood-resistant Bongkol (*Nauclea spp.*)
105 and other native saplings. Figure 1 shows areas of bare soil where clearing took place.
106 Within the restoration site a 50 m x 50 m botanical plot was also established, which we used to
107 compare drone- and field-derived ACD measurements. The project received permission to conduct
108 drone surveys and field data collection in Pin Supu from the Sabah Biodiversity Centre (SaBC) (access
109 license number JKM/MBS.1000-2/2 JLD.11 (11)).

##### 110 Fig 1. Orthomosaic of the Kaboi Lake restoration site.

111 Kaboi Lake is located in the Lower Kinabatangan Wildlife Sanctuary in eastern Sabah, Malaysia, at the
112 northern end of the Southeast Asian island of Borneo (see inset maps). Red line indicates the 2 ha
113 restoration site boundary; green line indicates the 50 m x 50 m botanical plot boundary.

## 114 Aboveground carbon density measurements from drone data

### 115 Drone data collection

116 We collected drone imagery of the Kaboi Lake site on 22^nd^ March 2021 using a DJI Phantom
117 4 Pro V2.0 quadcopter equipped with a 20 megapixel optical camera (DJI, Shenzhen, China). Flight
118 planning was conducted with a tablet and DroneDeploy planning software [(www.dronedeploy.com](http://www.dronedeploy.com/)).
119 The flights were fully autonomous and followed two ‘lawnmower’ patterns, overlapping at 90°, to
120 increase redundancy and reduce occlusions for the SfM processing [29]. Flight altitude was set at 70
121 m above ground level, resulting in a ground sampling distance of approximately 5 cm, with a flight
122 speed of 5 m s^-1^ and front and side image overlap of 80%. Three flights of ≈15 minutes each were
123 required to gather a total of 597 images for the 2 ha site.

### 124 Structure-from-motion processing of images

125 We performed all SfM image processing using OpenDroneMap (ODM) [39], an opensource
126 software ecosystem developed for processing aerial imagery. ODM utilises embedded Exchangeable
127 Image File Format (EXIF) tags within image files to access information on geolocation and camera
128 parameters. The processing pipeline in ODM consisted of five key processes and algorithms [40]:
129 structure-from-motion, producing a spare point cloud; multi-view stereo, generating a dense point
130 cloud; meshing, to create 3D polygonal surfaces from the point cloud; texturing, to then colour the
131 polygons using the relevant input images; and finally georeferencing, which transforms the local
132 coordinate system using geolocation data embedded in the
133 input images.
134 We conducted all processing on a desktop PC with an Intel Core i7 CPU and 16GB RAM,
135 although more memory is recommended for processing >200 images [40]. All ODM parameters were
136 left as default apart from the following two: input images were resized to a width of 4096 pixels
137 (from 4864) to decrease processing time whilst maintaining high resolution; and the minimum
138 number of features to be extracted from each image for matching in the SfM process was increased
139 from 8,000 to 28,000 due to the lack of distinguishable features in forest canopies. Processing 597 images took 3.5 hours.

### 140 Point cloud processing into canopy height models

141 Adapting the workflow outlined by Mlambo et al. [41], we post-processed the georeferenced
142 point cloud using the LAStools suite of LiDAR processing tools [42] in QGIS (version 3.14.16) [43].
143 Several steps were required to produce a digital elevation model (DEM), digital surface model (DSM),
144 and canopy height model (CHM) from the data, as outlined in Fig 2. Due to the file size limitations of
145 LAStools algorithms, the point cloud was first split into smaller tiles and then cleaned with the
146 *lasnoise* tool. *Lasnoise* identifies and removes isolated points that have few other points within a
147 three-dimensional search grid centred on that respective point. Cleaned points were then classified
148 as either ground or non-ground returns using *lasground* and *lasclassify*, tools developed for
149 extracting bare-earth points from airborne LiDAR data. The tiles were then thinned, with only the
150 highest points within a 0.05 m x 0.05 m grid (half the intended final resolution) being used to
151 generate DEM tiles, and with only the lowest points used for DSM tiles. Finally, the tiled DEM and
152 DSM rasters were merged to create a single DEM and DSM for the whole site, both at 0.1 m
153 resolution.

##### 154 Fig 2. Workflow for creating a canopy height model (CHM) from point cloud data.

155 The DEM produced in the previous step was very uneven, especially towards the edges of
156 the target site and in places where vegetation cover was high, which did not correspond with the
157 known minimal relief across the site. To resolve this issue, we produced a planar, flat DEM by taking
158 the 15^th^ percentile value of the original DEM as a proxy for the true ground elevation across the site.
159 We verified this assumption by examining the histogram of values for the original DEM and
160 confirming that the chosen ground elevation was a peak value – the most common elevation was
161 very likely to be the floodplain surface given the large areas of exposed ground at the site (Fig 1). This
162 approach has been previously used to generate DEMs in other biomass studies of similar tropical
163 forests with little relief, such as mangrove areas [25]. We created a CHM raster layer by subtracting
164 the flat DEM from the DSM (Fig 2), thereby normalizing the heights of the DSM.

### 165 Validating the canopy height model

166 We validated the CHM-derived height values by comparing them to field-measured tree heights within the botanical plot (field methods described below). Although the trees in the botanical plot had been surveyed, no geolocation information was recorded, preventing direct extraction of specific tree heights from the CHM. To overcome this, we located individual trees within the CHM using the Python package PyCrown [44]. PyCrown uses local maxima within the CHM to locate tree top positions and delineates tree crowns using region-growing algorithms adapted from [45]. We produced five different estimates of tree numbers and locations using various input parameters, as outlined in S1 Text. We used multiple estimates because field measurements could not be matched directly to the CHM, and different input parameters resulted in over- or underestimates of tree numbers in the botanical plot. Aside from those in S1 Text, all other PyCrown settings were left as default.

### 167 Error propagation in canopy height models

168 Biomass measurements from allometric equations are subject to various sources of
169 uncertainty, from model parameter estimates to field measurement errors. These
170 errors are thought to represent over 20% of the measured biomass at a plot level [46,47]. To account
171 for uncertainties in drone-derived measurements of biomass (and therefore carbon), we first
172 calculated the mean top-of-canopy height (TCH in meters), a key value for the plot-aggregate equations used
173 below, by averaging the pixels values within the CHM for the botanical plot. We propagated
174 uncertainty using the Monte Carlo method. Root mean square errors (RMSEs) associated with drone
175 measurements of canopy height can range from less than 0.5 m [48,49] to over 5 m [26,50], though
176 sparse ground coverage [51] and lower canopy heights (<24 m) [50,52] generally contribute to more
177 accurate results. Since the botanical plot had relatively small trees (<20 m) and large areas of bare
178 ground (leading to potentially more accurate measurements), we used two separate error
179 distributions to model different measurement error scenarios: one smaller error distribution with a
180 small standard deviation (σ = 1.5 m) and a more conservative distribution with larger errors (σ = 4 m).
181 1,000 values of mean TCH were generated using each error distribution, yielding 2,000 values for
182 mean TCH for the botanical plot.

### 183 Plot-aggregate allometric equations

184 From a literature review, we identified five suitable plot-aggregate allometric
185 equations to generate ACD measurements from the drone-derived CHM (Table 1).
186 Equations *I* [53] and *IV* [30] are simple power functions which suggest a relationship between canopies
187 height and ACD, and calculate ACD from mean TCH. Equation *I* was calibrated with data from
188 pantropical forests and equation *IV* was based on samples from peat swamp forests in Kalimantan. Equations
189 *II*, *III* [54] and *V* [18] are differently calibrated versions of an additional model developed by Asner
190 and Mascaro [53], in which ACD is measured using TCH as well as estimates of basal area (cross-
191 sectional area of all stems; BA in m^2^ ha^-1^) and wood density (WD in g cm^-3^). To apply these equations
192 to areas where measurements of basal area and wood density are not available, sub-models are
193 used to calculate BA and WD from TCH, meaning ACD can be measured using the single metric TCH.
194 Equations *II* and *III* were calculated by fitting data from 36 forest plots in Kabili-Sepilok Forest
195 Reserve, a remnant of old-growth tropical forest in eastern Sabah, to Asner and Mascaro’s [53]
196 generalised model. Equation *II* used sub-models to estimate BA and WD from TCH, while equation III
197 used field measurements instead (equations in Table 1 simplified by authors). Equation V was
198 calibrated using plot inventories from five forest reserves across the state of Sabah (including Kabili-
199 Sepilok Forest Reserve), and used sub-models to estimate BA and WD. We applied the five equations
200 to the 2,000 mean TCH values, resulting in 10,000 separate plot-aggregate ACD measurements for
201 the botanical plot, which were categorised by both the degree of error associated with height
202 measurements within the drone data, and by allometric equation.

##### 203 Table 1. Selected plot-aggregate aboveground carbon density (ACD) allometric equations for use

204 **with remotely-sensed height measurements.**

| **Equation** | **Forest type** | **Sample data range** | **ACD equation** | **Reference** |
| --- | --- | --- | --- | --- |
| *I* | Pantropical  forests | *n* plots = 754 | 𝐴𝐶𝐷 = 6.85 × 𝑇𝐶𝐻^0.952^ | [53] |
| *II* | Lowland tropical  rainforest, Sabah | *n* = 45,214;  *n* plots = 36; | 𝐴𝐶𝐷 = 7.37 × 𝑇𝐶𝐻^0.87^ | [54] |
|  |  | DBH range: 12-165 cm; |  |  |
|  |  | H range: 16-72 m |  |  |
| *III* | Lowland tropical  rainforest, Sabah | *n* = 45,214;  *n* plots = 36; | 𝐴𝐶𝐷 = 1.03 × 𝑇𝐶𝐻^1.535^ | [54] |
|  |  | DBH range: 12-165 cm; |  |  |
|  |  | H range: 16-72 m |  |  |
| *IV* | Peat swamp pole  forest, | *n* plots = 22 | 𝐴𝐶𝐷 = 0.47 × 𝑇𝐶𝐻^1.87^ | [30] |
|  | Kalimantan |  |  |  |
| *V* | Lowland tropical | *n* = 261,937; | 𝐴𝐶𝐷 = 0.567 × 𝑇𝐶𝐻^0.554^ | [18] |

rainforest, Sabah

*n* plots = 173

× 𝐵𝐴1.081

× 𝑊𝐷0.186 where *BA* = 1.112 x *TCH*, *WD* = 0.385 x *TCH*^0.097^

205 ACD in Mg C ha-1; TCH, mean top of canopy height in m; BA, stand basal area in m2 ha-1; WD,
206 community-weighted mean wood density in g cm-3. Forest types and underlying sample data ranges
207 are given where available. H, crown height in m; DBH, diameter at breast height in cm.

## 208 Aboveground carbon density measurements from field data

### 209 Field data collection

210 Field-based tree inventory data was collected for the 50 m x 50 m botanical plot (Fig 1) in
211 October 2021. The team recorded the boundaries of the restoration site and the botanical plot using
212 a Garmin GPSMAP 64s (± 3.7 m accuracy; Garmin, Olathe, USA). Diameter at breast height (DBH, in
213 cm) was measured for each tree (n = 24), as well as crown height (H, in m) using a clinometer and
214 tape measure. Wood density (WD, in g cm^-3^) was not directly measured, and field staff were
215 unable to identify trees to the species or genus level. This meant that wood density estimates could
216 not be obtained from species-specific databases, a common alternative to direct measurements in biomass studies [31]. Instead, we identified a range of plausible community mean WD values from published ecological studies of Southeast Asian rainforests [55–57], which informed the WD distributions used in the following error propagation steps.

### 217 Error propagation in field measurements

218 Adapting the workflow of Réjou-Méchain et al. [58], we propagated uncertainty in field-
219 based measurements of DBH and H using the Monte Carlo method. To calculate uncertainty
220 in WD, values were assigned from a normal distribution with a mean of 0.54 g cm^-3^ and
221 a standard deviation of 0.11 g cm^-3^. Using the above terms, we ran 1,000 simulations for each tree
222 within the plot (n = 24), resulting 1,000 sets of plot measurements.

### 223 Individual tree allometric equations

224 We used 27 different allometric equations to calculate the average ACD value for the
225 botanical plot using the field data (S1 Table). Since most community organizations lack the
226 capacity for direct sampling, we sought to understand the magnitude of over- or underestimation in
227 ACD values derived from preexisting equations not calibrated with on-site sampling or based on
228 different empirical datasets [31], necessitating a large selection of equations. We identified the 27
229 equations based on their applicability to the study site; they ranged in specificity from pantropical
230 moist forests to individual forest reserves. All site-specific equations derived from forests in
231 Borneo or the neighboring Indonesian island of Sumatra. Individual tree allometries calculate
232 aboveground biomass (AGB in kg). Plot level AGB values were converted to ACD by combining the AGB
233 values of all trees (n = 24) for each simulation, dividing by the plot area (0.25 ha), and using a
234 carbon content conversion factor of 0.47 [59]. This process resulted in a total of 27,000 ACD calculations for
235 the botanical plot.

# 236 Results

## 237 Structure-from-motion outputs

238 The DSM and initial DEM produced from the point cloud had a final resolution of 0.1 m x 0.1
239 m. The DEM showed a large variation in elevation across the restoration site (21.4 m) and within the
240 botanical plot (6.9 m; Fig 3). As mentioned previously, this variation did not correspond with the known
241 elevation profile of the site (<1 m). Height variations were more pronounced towards the edge of
242 the site and underneath denser vegetation and, though less prominent, also occurred in the
243 botanical plot.

##### 244 Fig 3. Digital elevation model of the restoration site generated from classified point cloud.

245 0.1 m resolution. Red line indicates the restoration site; green line indicates the botanical plot.
246 Elevation is significantly higher towards the perimeter of the sire due to poor canopy penetration in
247 the drone imagery.
248 Canopy height values for the normalized CHM (corrected using a planar DEM; Fig 4) ranged
249 from 0.38 m to 30.63 m. The mean TCH across the restoration site was 7.19 m (σ = 6.19 m; median = 5.72
250 m). Canopy height within the botanical plot had a much smaller range, from 0.20 m to 22.60 m, with
251 a mean TCH of 3.90 m (σ = 4.41 m; median = 2.01 m).

##### 252 Fig 4. Normalized canopy height model of the restoration site.

253 0.1 m resolution. Red line indicates the restoration site; green line indicates the botanical plot. A flat,
254 planar digital elevation model was used to normalise the point cloud-derived digital surface model.
255 Crown identification from our drone images required considerable field calibration. Figure
256 5A shows the locations of all tree crown tops >3 m found in the CHM by PyCrown, using estimate 5 (S1 Text) as an example. In Fig 5B, which focuses on the botanical plot, the grey lines
257 indicate the delineated boundaries of the tree crowns found using the same parameters. The crown
258 locations and extents identified in estimate 5 were generally accurate, albeit with some errors
259 towards the edges of the restoration site. This pattern was typical of all five estimates. The
260 field team identified 24 individual trees between 3 and 19 m high for analysis within the botanical
261 plot. None of the five estimates produced using PyCrown returned the same number of tree crowns
262 as the field team, with estimates ranging from 17 to 30 crowns. The crown heights derived from the
263 drone data were similar to those measured in the field (Fig 6). The mean and
264 median crown heights for the drone estimates ranged from 6.65 m to 8.25 m and 4.43 m to 5.81 m,
265 respectively, while the field measurements had a mean height of 8.16 m and a median of 7.25 m.
266 The drone estimates showed clear groupings of crowns <10 m, with fewer larger individuals.
267 A similar pattern was observed in the field measurements, although with a greater number of
268 crowns <13 m and only two crowns >15 m (Fig 6).

##### 269 Fig 5. Location and extent of tree crowns within the restoration site.

270 Tree crowns identified using PyCrown; figure shows results of PyCrown estimate 5. (A) Location of all
271 tree crowns >3 m tall within the restoration site. (B) Location and extent of tree crowns >3 m tall
272 within the botanical plot.

##### 273 Fig 6. Field- and drone-derived individual tree crown height measurements.

274 Samples 1-5 are measurements extracted from the canopy height model using different input
275 parameters for PyCrown. Height measurements from field data shown in orange. Number of
276 individual tree crowns >3 m identified by each sample is shown at the bottom.

## 277 Aboveground carbon density measurements from drone data

278 Drone-derived estimates of biomass have significantly higher uncertainty compared to those based on field data. The distribution of ACD measurements for the botanical plot produced using five different plot-aggregate equations (Table 1) are shown in Fig 7. For comparison, Figure 7 also shows the
279 combined distribution of all field-derived ACD measurements using 27 different allometric
280 equations (S1 Table). Across all five drone-derived distributions, a fivefold variation in mean
281 and median ACD values was observed. The ACD values calculated using the larger modelled height measurement
282 errors (σ = 4 m; Fig 7B) showed substantial differences in distribution ranges. The variation within the
283 measurements for each equation was significantly greater with larger height measurement errors
284 compared to the smaller errors (σ = 1.5 m; Fig 7A).

##### 285 Fig 7. Distributions of field- and drone-derived aboveground carbon density (ACD) values for the

286 **botanical plot.**
287 For drone data, combined ACD values for all five allometric equations are shown in dark green, with
288 individual equations in light green. For field data, combined ACD values from 27 allometric equations
289 are shown in orange. (A) ACD distributions calculated using small modelled errors in drone height
290 measurements (σ = 1.5 m). (B) ACD distributions using large modelled errors (σ = 4 m).
291 With larger errors, the combined mean ACD value for all five equations was 16.78 Mg C
292 ha^-1^ (σ = 17.79 Mg C ha^-1^), compared to a field-derived mean ACD value of 6.05 Mg C ha^-1^ (σ =
293 2.07 Mg C ha^-1^; all 27 equations) (Fig 7B). For smaller error estimates, the mean ACD was 14.06 Mg C
294 ha^-1^ (σ = 10.64 Mg C ha^-1^) (Fig 7A). There was a clear difference between the measurements
295 produced by equations *I* and *II*, and equations *III*-*V*. Under both measurement error scenarios,
296 equations *I* and *II* produced mean ACD values approximately six times higher than those derived from field data. The
297 mean ACD values for equations *III*-*V* were lower, and those using smaller measurement errors more
298 closely resembled field measurements. When equations *III*-*V* were combined, the mean ACD value
299 was 7.19 Mg C ha^-1^ (σ = 4.68 Mg C ha^-1^) with smaller errors, and 10.95 Mg C ha^-1^ (σ = 13.20 Mg
300 C ha^-1^) with larger errors. However, the range of ACD values for equations *III*-*V* exceeded that of the
301 field measurements under both error distributions.
302 When applying the plot-aggregate equations across the whole restoration site and averaging
303 the results, the carbon density value was twice that of the botanical plot. Using the smaller height
304 error distribution, mean ACD was 29.28 Mg C ha^-1^ (σ = 13.61 Mg C ha^-1^), and using large errors it was
305 31.27 Mg C ha^-1^ (σ = 22.63 Mg C ha^-1^). When just equations *III*-*V* were combined, mean ACD values
306 were 20.24 Mg C ha^-1^ (σ = 7.50 Mg C ha^-1^) using small errors and 23.95 Mg C ha^-1^ (σ = 20.67 Mg C ha^-1^)
307 using large errors.

# 308 Discussion

## 309 Aboveground carbon density measurements

310 Drone-based ACD calculations for our field plots were systematically higher than field-
312 based measurements and had wider uncertainties (Fig 7). The mean drone-derived ACD
313 measurements for the plot were approximately double the field-based carbon density,
314 which we assume is a true-to-reality benchmark. Two commonly used pantropical allometric equations,
315 equations 1 [60] and 20 [61] in S1 Table, frequently serve as ‘general allometric equations’
316 in individual tree AGB studies [31,62–64] or as the basis for new allometric models [18,54]. These equations produced ACD distributions either side of the mean field-derived ACD value from all 27 equations. This increased our confidence that the distribution of ACD values across the 27 equations represented a plausible range which contained the true ACD value for the plot, making it suitable for comparison with the drone measurements.
317 Three of the drone-derived values (equations *III-V*) were more similar to the field-based
318 values, albeit with greater variability. A key factor here is the underlying
319 datasets for these equations: all were calibrated using field plots that share general
320 geographical and ecological similarities with Kaboi Lake. In contrast, the generalised pantropical
321 allometric equation *I* was developed using primarily Neo- and Afrotropical forest plots, which are
322 structurally distinct from Borneo’s forests [65]). While equations *II* and *III* were both
323 derived from Sepilok-Kabili Forest Reserve, equation *II* used sub-models to
324 predict diameter at breast height and wood density, whilst *III* used field measurements. Both
325 equations *I* and *II s=*overestimated carbon densities for the plot by a greater degree. These results
326 indicate that the selection of allometric equations significantly influences the accuracy of ACD calculations from SfM data. However, drone-derived SfM can be a viable method for producing ACD values comparable to field-based methods at a community scale, provided regionally calibrated equations are used.
327 Differences in calculation methods and assumptions between the field- and drone-based
328 approaches may explain the observed bias towards larger drone-derived ACD values. ITC
329 approaches, like our field-based methods, calculate carbon within discrete units (individual
330 trees), excluding smaller trees (those <3 m), low-lying
331 vegetation, and deadfall is not included in total carbon density calculations. In contrast, the plot-
332 aggregate method used in this study did not differentiate between trees and non-trees, and included
333 all biomass within the CHM when calculating mean TCH. While this theoretically results in higher carbon
334 values, shorter trees and vegetation have a disproportionately small impact on total
335 carbon in practice. Differences may also arise from large tree crowns that cross the plot boundary. These trees
336 were not recorded by the field team as their trunks lay outside of the boundary but, due to the
337 ‘cookie cutter’ methods used to extract values from the CHM, they did contribute to the overall
338 carbon values calculated via plot-aggregate approach. These edge effects were perhaps amplified by
339 the small relative size of the plot [53]. Differences may also arise from uncertainties in the drone-
340 derived CHM, which are discussed below.
341 Our calculated ACD values for Kaboi Lake are significantly lower than other published values
342 for secondary forests in Borneo. Previously logged forests in Sabah can contain carbon densities of
343 60-140 Mg C ha^-1^ [15], whilst for secondary peat forests in Kalimantan, ACD ranges from 40-100 Mg C
344 ha^-1^ [30,66]. These values are approximately an order of magnitude greater than those measured at
345 the botanical plot. The low carbon density at Kaboi Lake could feasibly be explained by both historic
346 logging of dipterocarps and the recent clearing, and Asner et al. [15] show that recently deforested
347 lands in Sabah (<5 years) have significantly lower carbon densities (7 Mg C ha^-1^), more consistent
348 with our results.
349 Differences between our results and other published ACD values for secondary forests suggest
350 the potential for overestimation of baseline carbon density values at
351 restoration sites, especially if using remotely sensed imagery with low resolution relative to site size. The
352 drone-based methods we outline here offer a more accurately solution for assessing
353 baseline carbon values for community-scale ACD measurements compared to
354 satellite-derived methods. Further, the five plot-aggregated allometric equations (Table 1) were not
355 necessarily developed and calibrated for use in severely degraded forest. The future use of drone
356 SfM and plot-aggregate allometries specifically calibrated for severely degraded forest may reveal
357 further differences between assumptions used in restoration planning and carbon accounting, and
358 on-the-ground ACD values.

## 359 Methodological limitations and uncertainties

360 Uncertainties in the drone-derived ACD values arise from both the selection of allometric
361 models and generation of the CHM. Mean ACD measurements varied by a factor of 4 between
362 equations using the smaller height measurement errors, and by a factor of 3 when using larger errors
363 (Fig 7). Clear groupings emerged among the equations, with equations *III*-*V* more closely matching
364 field-derived measurements. This grouping is explained by the difference in underlying datasets used
365 to produce the equations, highlighting the importance of equation selection for this method.
366 However, within all individual plot-aggregate equations exhibited a much broader
367 distribution of results compared to field measurements, reflecting the height measurement errors
368 associated with drones. This broader distribution was caused by the size of the error distributions used to propagate
369 uncertainty in the mean TCH values relative to the CHM height. The mean TCH value for the
370 botanical plot was 3.9 m, while the error distributions had standard deviations of 1.5 m and 4 m.
371 Using ground control points (GCPs) in the data collection phase could reduce the uncertainties
372 surrounding drone height measurements [48,50], but Fig 7A shows that even with the reduced errors
373 expected from GCP correction (i.e., modelled using the smaller error distribution), large uncertainties
374 in ACD measurements remain.
375 The accuracy of the canopy height model is ultimately dependent on the digital surface and
376 elevation models generated by SfM, with DEMs having a greater impact on accuracy due to the
377 relative size of their measurement errors. Limited canopy penetration with optical imagery poses a challenge
378 for SfM, resulting in fewer ground returns and poorer quality DEMs compared
379 to LiDAR data [26,51,67–69]. Nevertheless, DEMs derived from optical drone imagery have been
380 successfully used to measure forest biomass [25,70], especially in woodlands with relatively open
381 canopies [71], similar to our study site. Although Kaboi Lake had visible bare ground, we
382 achieved higher accuracy in our CHM by assuming a flat, low relief surface rather than using the DEM
383 produced by SfM, which included a relief of 21.4 m. This approach is not feasible in regions of
384 significant topographic relief or complex topography. Nevertheless, it avoids the
385 issues of matching datasets from different sensors and platforms, making it a
386 plausible technique for minimising errors in SfM-derived DEMs and CHMs, particularly
387 when drone imagery is available during the pre-restoration forest clearance.
388 Ground control points (GCPs) are usually an important part of the SfM workflow, used to
389 accurately locate, orient and scale point clouds in space [72]. However, we experienced technical
390 issues in the acquisition and integration of GCPs into the ODM software. Hence, we analysed the
391 data without ground controls and examined the impact of omitting this data collection process. We
392 used only the drone’s onboard global navigation satellite system (GNSS) receiver to provide
393 geospatial data and scale the CHM, and used a comparison of tree heights from field measurements
394 and the CHM to validate the scaling. The tree crown heights extracted using PyCrown followed
395 similar distribution patterns to the field measurements, with the majority of individual crowns
396 measuring <10 m across all measurements (Fig 6). However, clear differences emerged in the
397 number of tree crowns identified in the botanical plot across PyCrown estimates. Increased numbers
398 of taller trees (>10 m) identified within the plot may be explained by the presence of large,
399 overhanging canopies from trees that are situated outside of the botanical plot.
400 The maximum field-measured crown height was 18.8 m, and omitting the (presumed
401 overhanging) trees taller than 18.8 m from estimates 1-4 produces distributions more closely aligned with
402 the field measurements but also reduces mean heights. The discrepancy in mean heights may be
403 due to the downscaling of the CHM for PyCrown processing, which reduces the ‘visibility’ of
404 fine-scale canopy peaks [73,74] and thereby reduces height measurements. The lower
405 mean crown heights also follow other results showing a systematic underestimation of TCH using
406 SfM [26,32,68,75]. However, additional studies have demonstrated SfM overestimating TCH in open
407 canopy forest [74], or the biases shift changes I canopy height [76]. As this study
408 utilised a flat DEM, it negated the impact of ground occlusion in the DEM which is often a major
409 contributor to reported underestimations of canopy height. Of importance, then, is the fact that
410 errors in field measurement methods were not considered in these comparisons and are another
411 potential source of bias. Canopy height is the key uncertainty in field measurements; DGFC staff
412 estimated uncertainty in canopy height measurements at approximately 3 m, exacerbated by taller
413 trees or the use of novice surveyors. Despite differences between the sets of measurements, the
414 coincident uncertainties between field and drone-derived data suggest that the CHM was scaled
415 sufficiently during the SfM process to enable plausible ACD measurements to be produced, as the
416 uncertainties here were smaller than those associated with allometric equation selection.

## 417 Implications of method for community-scale carbon monitoring

418 Our findings suggest that lightweight, low-cost, consumer-grade drones combined with
419 open-source software presents a viable solution for generating ACD values within community-scale
420 projects. There is an optimal scale for using drones for ACD measurements with regards to trade-offs
421 between accuracy, simplicity, and cost-effectiveness. This optimal scale ranges between individual plot-level
422 and regional-scale surveys, i.e., between approximately 1 and 100 ha. Between these bounds,
423 drones offer an attractive option for data acquisition and carbon measurement, aligning well with
424 the needs of community-scale ACD monitoring while bridging the gap between field-based and satellite-
425 based measurements.
426 At scales between 1 and 100 ha, drone derived ACD estimates can be obtained without
427 extensive field surveys and using only a single input metric. Our findings futher support the idea that
428 drones offer a fast and cost-effective option for data acquisition at
429 scales of up to tens of hectares [29,33,77]. A team of two people were able to map the entire 2 ha
430 restoration plot art a high resolution (5 cm) in a single morning, whereas collecting field-
431 based measurements for each tree in the same plot would take two people several days. Due to
432 the reduction in survey time per unit area surveyed, the drone-based method we demonstrate here
433 is a promising option for scaling up carbon monitoring from a botanical plot level. For exemple, canopy height
434 metrics for a 10 ha site can be measured using drones more quickly than gathering field
435 measurements for a single 0.25-1 ha plot. While field plots remain necessary for
436 calibration and verification, this approach significantly reduces total survey times.
437 However, at smaller scales (<2 ha) and with one-off surveys, it is worth recognising that it
438 may be simpler, faster, and cheaper to utilise field-based methods over drone-based SfM. Although
439 field-based methods do require more input metrics and require certain surveying skills, they do not
440 require training in piloting and data processing, nor the purchase of comparatively expensive
441 hardware – the drone used here cost approximately £1,500 (field staff already had access to a
442 smartphone for mission planning). Still, with larger areas or repeat surveys, the simplicity and
443 potential accuracy benefits of field-based methods may be outweighed by the subsequent financial
444 advantages (e.g., reduced labor costs) of drone-based SfM.
445 Drone use encounters practical limitations at larger scales. The
446 high temporal and spatial resolution of drone imagery allows for better detection of forest
447 structure than freely available imagery that could be used for larger-scale (>100 ha) ACD
448 measurements (e.g., Landsat or ESA’s CCI biomass dataset). Whilst drone-based SfM has been used
449 over these scales [26], there are potential trade-offs between resolution, extent and labour costs
450 (greater spatial resolution may require more, lower altitude flights). The relatively short range of
451 drones also introduces issues concerning travelling to launch sites, both in terms of accessibility and
452 total survey times. For surveys >100ha, purchasing high-resolution (30 cm) snapshot satellite imagery
453 for a site, or even commissioning an airborne LiDAR survey, may become a more practical option
454 (e.g., a WorldView-3 satellite image encompassing the site would have cost ≈£400). These
455 approaches do, however, come with disadvantages related to temporal resolution and repeatability,
456 and would still require field-based measurements of ACD within botanical plots to calibrate
457 imagery.
458 Although the methods we demonstrate have many potential advantages for community-
459 scale carbon monitoring, there are several factors that may limit the method’s accessibility for
460 community use. First is the need for, access to, and costs of pilot training. Piloting a multirotor drone
461 may be straightforward, but precise flight planning is required to maximise the accuracy of any SfM
462 outputs. Variables such as sun angle during image capture, camera angle, and image
463 overlap significantly affect point cloud construction [29,78]. A few days of training should be sufficient to
464 pilot a multirotor safely, set up and record GCPs, and collect imagery suitable for SfM, though more
465 training may be needed for fixed wing drones.
466 Second is the role of data processing; it is easy to focus on flying a drone, but this is only half
467 the process of producing ACD measurements. Any community-scale groups or actors wishing to
468 replicate these methods will need a good working knowledge of GIS, Python and relevant
469 opensource software, such as ODM. This, again, may require additional training. Data processing also
470 takes a considerable time; processing ~600 images and producing point clouds took over 3 hours on
471 a powerful desktop PC. Added to this are the multiple attempts over several days that failed part way
472 through due to insufficient memory. In combination, lengthy data processing steps may additionally
473 reduce time advantages over manual field sampling (albeit less so for larger sites).
474 Finally, there are considerable additional expenses beyond just purchasing a drone. A laptop
475 capable of running the SfM and data processing software may cost as much as the
476 drone itself (up to approximately £1,000). However, like a drone, its applicability for other purposes
477 may counterbalance these additional costs. A tablet is required to operate the drone, although
478 smartphones, which can also be used, are becoming increasingly common even in rural areas. Surveys with
479 consumer-grade drones often require additional hardware, such as handheld GNSS receivers for
480 recording GCPs (≈£300 for a basic unit), and paid subscriptions to photogrammetry software
481 (PIX4Dmapper, a popular photogrammetry program, currently costs ≈£220 per month;
482 [www.pix4d.com](http://www.pix4d.com/)). As demonstrated in this study, open-source photogrammetry software can reduce costs, as
483 can forgoing GCPs and using geolocation data
484 embedded in the input images. Additionally, there are the costs associated with obtaining permits or certificates
485 required to fly in the region. The costs here may be small, but the legislation introduces an additional
486 potential barrier, as community groups may find navigating the myriad forms and administrative
487 requirements more difficult than academics with connections to local universities and
488 forestry departments.
489 These considerations apply not only to the use of drones for carbon monitoring, but also
490 they be limited to the use of drones in Borneo. Drones will always interact with real-
491 world factors that can limit the accessibility of such methods. Conservation areas differ significantly
492 from controlled environments like testing laboratories or university campuses and can present
493 unexpected challenges [79]. In our case, extreme temperatures limited the duration of drone
494 surveys, whilst routine flooding delayed data collection for several months. It is worth
495 considering how these environmental factors might affect the practical use of
496 other conservation and remote sensing technologies. Additionally, overlooked factors like
497 species identification skills or data-handling capacities may limit other participatory monitoring
498 approaches, even when drones are not involved. Awareness of these factors is important for managing
499 expectations when using new remote sensing technologies to address environmental questions, not only in Borneo, but in forest ecosystems and conservation spaces in general.

# 500 Conclusions

501 In this paper, we developed, applied, and analysed a new method for incorporating
502 consumer-grade drones into community-scale aboveground carbon measurements, utilising
503 opensource software, drone-derived SfM, and pre-published plot-aggregate allometric equations.
504 Our results show that this method presents a viable option for generationg ACD measurements for
505 community-scale conservation and restoration projects, producing results comparable to those
506 obtarined using established field-based methods. Drone-derived measurements were
507 larger than field-derived measurements, but varied depending on the allometric models used. This highlights the
508 importance of selecting regionally calibrated allometric equations when applying this
509 method. The development of new models for a range of forest types across the tropics will
510 greatly increase this method’s accuracy and applicability.
511 The approach presented here offers several advantages over existing methodologies that could
512 be used for community-scale ACD measurements, including a reduction in survey times and long-
513 term costs. However, several factors may limit the accessibility of this method
514 for community groups in practice. These barriers - analogous to those in other methodologies, technologies, and location – may be resolved with relative ease, but should not be overlooked. Nevertheless, the method described here has established a foundation for a simple drone-based workflow to measure carbon, showing promise for real-world applications and potential refinement in future studies.

# 515 Acknowledgements

516 We are grateful to the Sabah Biodiversity Centre and the Sabah Wildlife Department for permissions
517 to carry out the research and for collaboration with the project. We also wish to thank KOPEL Bhd,
518 Regrow Borneo, and research assistants at the Danau Girang Field Centre for their efforts in
519 collecting field data and their activities at the restoration site.

# 520 References

521 1.	Tyukavina A, Baccini A, Hansen MC, Potapov P V., Stehman S V., Houghton RA, et al.
522 Corrigendum: Aboveground carbon loss in natural and managed tropical forests from 2000 to

554	2012 (2015 Environ. Res. Lett. 10 074002). Environ Res Lett. 2018;13: 109501.

555	2.	Pearson TRH, Brown S, Murray L, Sidman G. Greenhouse gas emissions from tropical forest

556	degradation: an underestimated source. Carbon Balance Manag. 2017;12: 3.

557	doi:10.1186/s13021-017-0072-2

558 3.	Bastin J-F, Finegold Y, Garcia C, Mollicone D, Rezende M, Routh D, et al. The global tree
559 restoration potential. Science (80- ). 2019;365: 76–79. doi:10.1126/science.aax0848
560 4.	Olsson L, Barbosa H, Bhadwal S, Cowie A, Delusca K, Flores-Renteria D, et al. Land
561 Degradation. In: Shukla PR, Skea J, Calvo Buendia E, Masson-Delmotte V, Pörtner H-O,
562 Roberts DC, et al., editors. Climate Change and Land: an IPCC special report on climate
563 change, desertification, land degradation, sustainable land management, food security, and
564 greenhouse gas fluxes in terrestrial ecosystems. 2019.
565 5.	Erbaugh JT, Pradhan N, Adams J, Oldekop JA, Agrawal A, Brockington D, et al. Global forest
566 restoration and the importance of prioritizing local communities. Nat Ecol Evol. 2020;4:

567	1472–1476. doi:10.1038/s41559-020-01282-2

568	6.	Oldekop JA, Sims KRE, Karna BK, Whittingham MJ, Agrawal A. Reductions in deforestation and

569	poverty from decentralized forest management in Nepal. Nat Sustain. 2019;2: 421–428.

570	doi:10.1038/s41893-019-0277-3

571	7.	Persha L, Agrawal A, Chhatre A. Social and Ecological Synergy: Local Rulemaking, Forest

572	Livelihoods, and Biodiversity Conservation. Science (80- ). 2011;331: 1606–1608.

573	doi:10.1126/science.1199343

574	8.	Dawson NM, Coolsaet B, Sterling EJ, Loveridge R, Gross-Camp ND, Wongbusarakum S, et al.

575	The role of Indigenous peoples and local communities in effective and equitable

576	conservation. Ecol Soc. 2021;26: art19. doi:10.5751/ES-12625-260319

577	9.	Seymour F. Seeing the Forests as well as the (Trillion) Trees in Corporate Climate Strategies.

578	One Earth. 2020;2: 390–393. doi:10.1016/j.oneear.2020.05.006

579 10.	Di Sacco A, Hardwick KA, Blakesley D, Brancalion PHS, Breman E, Cecilio Rebola L, et al. Ten
580 golden rules for reforestation to optimize carbon sequestration, biodiversity recovery and
581 livelihood benefits. Glob Chang Biol. 2021;27: 1328–1348. doi:10.1111/gcb.15498
582 11.	Daguma L, Minang P, Aynekulu E, Carsan S, Nzyoka J, Bah A, et al. From Tree Planting to Tree
583 Growing: Rethinking Ecosystem Restoration. 2020. Report No.: ICRAF Working Paper No 304.

584	do[i:htt](http://dx.doi.org/10.5716/WP20001.PDF)p:[//dx.doi.org/10.5716/WP20001.PDF](http://dx.doi.org/10.5716/WP20001.PDF)

585 12.	Fleischman F, Basant S, Chhatre A, Coleman EA, Fischer HW, Gupta D, et al. Pitfalls of Tree
586 Planting Show Why We Need People-Centered Natural Climate Solutions. Bioscience. 2020.
587 doi:10.1093/biosci/biaa094
588 13.	Busch J, Engelmann J, Cook-Patton SC, Griscom BW, Kroeger T, Possingham H, et al. Potential
589 for low-cost carbon dioxide removal through tropical reforestation. Nat Clim Chang. 2019;9:

590	463–466. doi:10.1038/s41558-019-0485-x

591	14.	Galante MV, Pinard MA, Mencuccini M. Estimating carbon avoided from the implementation

592	of reduced-impact logging in Sabah, Malaysia. Int For Rev. 2018;20: 58–78.

593	doi:10.1505/146554818822824192

594	15.	Asner GP, Brodrick PG, Philipson CD, Vaughn NR, Martin RE, Knapp DE, et al. Mapped

595	aboveground carbon stocks to advance forest conservation and recovery in Malaysian

596	Borneo. Biol Conserv. 2018;217: 289–310. doi:10.1016/j.biocon.2017.10.020

597	16.	Csillik O, Kumar P, Mascaro J, O’Shea T, Asner GP. Monitoring tropical forest carbon stocks

598	and emissions using Planet satellite data. Sci Rep. 2019;9: 17831. doi:10.1038/s41598-019-

599	54386-6

600	17.	Gonçalves F, Treuhaft R, Law B, Almeida A, Walker W, Baccini A, et al. Estimating

601	Aboveground Biomass in Tropical Forests: Field Methods and Error Analysis for the

602	Calibration of Remote Sensing Observations. Remote Sens. 2017;9: 47.

603	doi:10.3390/rs9010047

604	18.	Jucker T, Asner GP, Dalponte M, Brodrick PG, Philipson CD, Vaughn NR, et al. Estimating

605	aboveground carbon density and its uncertainty in Borneo’s structurally complex tropical

606	forests using airborne laser scanning. Biogeosciences. 2018;15: 3811–3830. doi:10.5194/bg-

607	15-3811-2018

608	19.	Ruwaimana M, Satyanarayana B, Otero V, Muslim AM, Muhammad Syafiq A, Ibrahim S, et al.

609	The advantages of using drones over space-borne imagery in the mapping of mangrove

610	forests. PLoS One. 2018;13: e0200288. doi:10.1371/journal.pone.0200288

611	20.	Nowak MM, Dziób K, Bogawski P. Unmanned Aerial Vehicles (UAVs) in environmental biology:

612	A review. Eur J Ecol. 2019;4: 56–74. doi:10.2478/eje-2018-0012

613	21.	Chabot D, Hodgson AJ, Hodgson JC, Anderson K. ‘Drone’: technically correct, popularly

614	accepted, socially acceptable. Drone Syst Appl. 2022;10: 399–405. doi:10.1139/dsa-2022-

615	0041

616 22.	Cranston KA, Wong WY, Knowlton S, Bennett C, Rivadeneira S. Five psychological principles of
617 codesigning conservation with (not for) communities. Zoo Biol. 2022. doi:10.1002/zoo.21725
618 23.	Beaudoin G, Rafanoharana S, Boissière M, Wijaya A, Wardhana W. Completing the Picture:
619 Importance of Considering Participatory Mapping for REDD+ Measurement, Reporting and
620 Verification (MRV). Bond-Lamberty B, editor. PLoS One. 2016;11: e0166592.

621	doi:10.1371/journal.pone.0166592

622	24.	Westoby MJ, Brasington J, Glasser NF, Hambrey MJ, Reynolds JM. “Structure-from-Motion”

623	photogrammetry: A low-cost, effective tool for geoscience applications. Geomorphology.

624	2012;179: 300–314. doi:10.1016/j.geomorph.2012.08.021

625	25.	Otero V, Van De Kerchove R, Satyanarayana B, Martínez-Espinosa C, Fisol MA Bin, Ibrahim MR

626	Bin, et al. Managing mangrove forests from the sky: Forest inventory using field data and

627	Unmanned Aerial Vehicle (UAV) imagery in the Matang Mangrove Forest Reserve, peninsular

628	Malaysia. For Ecol Manage. 2018;411. doi:10.1016/j.foreco.2017.12.049

629	26.	Swinfield T, Lindsell JA, Williams J V., Harrison RD, Agustiono, Habibi, et al. Accurate

630	Measurement of Tropical Forest Canopy Heights and Aboveground Carbon Using Structure

631	From Motion. Remote Sens. 2019;11: 928. doi:10.3390/rs11080928

632	27.	Miller E, Dandois J, Detto M, Hall J. Drones as a Tool for Monoculture Plantation Assessment

633	in the Steepland Tropics. Forests. 2017;8: 168. doi:10.3390/f8050168

634	28.	Smith MW, Carrivick JL, Quincey DJ. Structure from motion photogrammetry in physical

635	geography. Prog Phys Geogr. 2016;40: 247–275. doi:10.1177/0309133315615805

636	29.	Iglhaut J, Cabo C, Puliti S, Piermattei L, O’Connor J, Rosette J. Structure from Motion

637	Photogrammetry in Forestry: a Review. Curr For Reports. 2019;5: 155–168.

638	doi:10.1007/s40725-019-00094-3

639	30.	Ferraz A, Saatchi S, Xu L, Hagen S, Chave J, Yu Y, et al. Carbon storage potential in degraded

640	forests of Kalimantan, Indonesia. Environ Res Lett. 2018;13: 095001. doi:10.1088/1748-

641	9326/aad782

642	31.	Yuen JQ, Fung T, Ziegler AD. Review of allometric equations for major land covers in SE Asia:

643	Uncertainty and implications for above- and below-ground carbon estimates. For Ecol

644	Manage. 2016;360: 323–340. doi:10.1016/j.foreco.2015.09.016

645	32.	Zahawi RA, Dandois JP, Holl KD, Nadwodny D, Reid JL, Ellis EC. Using lightweight unmanned

646	aerial vehicles to monitor tropical forest recovery. Biol Conserv. 2015;186: 287–295.

647	doi:10.1016/j.biocon.2015.03.031

648	33.	Puliti S, Solberg S, Granhus A. Use of UAV Photogrammetric Data for Estimation of Biophysical

649	Properties in Forest Stands Under Regeneration. Remote Sens. 2019;11: 233.

650	doi:10.3390/rs11030233

651	34.	Climate-data.org. Climate Batu Puteh. 2022 [cited 17 Mar 2022]. Available:

652	https://en.climate-data.org/asia/malaysia/sabah/batu-puteh-760259/

653	35.	Berry NJ, Phillips OL, Ong RC, Hamer KC. Impacts of selective logging on tree diversity across a

654	rainforest landscape: the importance of spatial scale. Landsc Ecol. 2008;23: 915–929.

655	doi:10.1007/s10980-008-9248-1

656	36.	Avitabile V, Herold M, Heuvelink GBM, Lewis SL, Phillips OL, Asner GP, et al. An integrated

657	pan-tropical biomass map using multiple reference datasets. Glob Chang Biol. 2016;22: 1406–

658	1420. doi:10.1111/gcb.13139

659	37.	SFD. 2nd Forest Management Plan (Pin-Supu Forest Reserve, Kinabatangan) (2018-2027).

660	Sandakan; Available: https://pinsupu.sabah.gov.my/images/publication/3.2-fmp-pinsupufr-

661	2018-2027.pdf

662 38.	SFD. Pin Supu Forest Reserve. 2020 [cited 17 Mar 2022]. Available:
663 <http://www.forest.sabah.gov.my/pinsupu/>
664 39.	OpenDroneMap Authors. OpenDroneMap. 2020. Available:
665 https://github.com/OpenDroneMap/ODM
666 40.	Toffanin P. OpenDroneMap: The Missing Guide. St Petersburg, FL: MasseranoLabs LLC; 2019.
667 41.	Mlambo R, Woodhouse I, Gerard F, Anderson K. Structure from Motion (SfM)
668 Photogrammetry with Drone Data: A Low Cost Method for Monitoring Greenhouse Gas
669 Emissions from Forests in Developing Countries. Forests. 2017;8: 68. doi:10.3390/f8030068
670 42.	rapidlasso GmbH. LAStools. Available: https://rapidlasso.com/lastools/
671 43.	QGIS Development Team. QGIS Geographic Information System. 2021. Available:
672 https://qgis.org/en/site/
673 44.	Zörner J, Dymond J, Shepherd J, Jolly B. PyCrown - Fast raster-based individual tree
674 segementation for LiDAR data. Landcare Research NZ Ltd; 2018.
675 doi:https://doi.org/10.7931/M0SR-DN55
676 45.	Dalponte M, Coomes DA. Tree-centric mapping of forest carbon density from airborne laser
677 scanning and hyperspectral data. Murrell D, editor. Methods Ecol Evol. 2016;7: 1236–1245.

678	doi:10.1111/2041-210X.12575

679	46.	Chave J, Condit R, Aguilar S, Hernandez A, Lao S, Perez R. Error propagation and scaling for

680	tropical forest biomass estimates. Malhi Y, Phillips OL, editors. Philos Trans R Soc London Ser

681	B Biol Sci. 2004;359: 409–420. doi:10.1098/rstb.2003.1425

682	47.	Chen Q, Vaglio Laurin G, Valentini R. Uncertainty of remotely sensed aboveground biomass

683	over an African tropical forest: Propagating errors from trees to plots to pixels. Remote Sens

684	Environ. 2015;160: 134–143. doi:10.1016/j.rse.2015.01.009

685	48.	Ganz S, Käber Y, Adler P. Measuring Tree Height with Remote Sensing—A Comparison of

686	Photogrammetric and LiDAR Data with Different Field Measurements. Forests. 2019;10: 694.

687	doi:10.3390/f10080694

688	49.	Birdal AC, Avdan U, Türk T. Estimating tree heights with images from an unmanned aerial

689	vehicle. Geomatics, Nat Hazards Risk. 2017;8: 1144–1156.

690	doi:10.1080/19475705.2017.1300608

691	50.	Kameyama S, Sugiura K. Estimating Tree Height and Volume Using Unmanned Aerial Vehicle

692	Photography and SfM Technology, with Verification of Result Accuracy. Drones. 2020;4: 19.

693	doi:10.3390/drones4020019

694 51.	Wallace L, Lucieer A, Malenovský Z, Turner D, Vopěnka P. Assessment of Forest Structure
695 Using Two UAV Techniques: A Comparison of Airborne Laser Scanning and Structure from
696 Motion (SfM) Point Clouds. Forests. 2016;7: 62. doi:10.3390/f7030062
697 52.	Iizuka K, Yonehara T, Itoh M, Kosugi Y. Estimating Tree Height and Diameter at Breast Height
698 (DBH) from Digital Surface Models and Orthophotos Obtained with an Unmanned Aerial
699 System for a Japanese Cypress (Chamaecyparis obtusa) Forest. Remote Sens. 2018;10: 13.

700	doi:10.3390/rs10010013

701	53.	Asner GP, Mascaro J. Mapping tropical forest carbon: Calibrating plot estimates to a simple

702	LiDAR metric. Remote Sens Environ. 2014;140: 614–624. doi:10.1016/j.rse.2013.09.023

703 54.	Coomes DA, Dalponte M, Jucker T, Asner GP, Banin LF, Burslem DFRP, et al. Area-based vs
704 tree-centric approaches to mapping forest carbon in Southeast Asian forests from airborne
705 laser scanning data. Remote Sens Environ. 2017;194: 77–88. doi:10.1016/j.rse.2017.03.017
706 55.	Morel AC, Saatchi SS, Malhi Y, Berry NJ, Banin L, Burslem D, et al. Estimating aboveground
707 biomass in forest and oil palm plantation in Sabah, Malaysian Borneo using ALOS PALSAR

708	data. For Ecol Manage. 2011;262: 1786–1798. doi:10.1016/j.foreco.2011.07.008

709	56.	Manuri S, Brack C, Nugroho NP, Hergoualc’h K, Novita N, Dotzauer H, et al. Tree biomass

710	equations for tropical peat swamp forest ecosystems in Indonesia. For Ecol Manage.

711	2014;334: 241–253. doi:10.1016/j.foreco.2014.08.031

712	57.	Kenzo T, Ichie T, Hattori D, Itioka T, Handa C, Ohkubo T, et al. Development of allometric

713	relationships for accurate estimation of above- and below-ground biomass in tropical

714	secondary forests in Sarawak, Malaysia. J Trop Ecol. 2009;25: 371–386.

715	doi:10.1017/S0266467409006129

716	58.	Réjou-Méchain M, Tanguy A, Piponiot C, Chave J, Hérault B. BIOMASS: an R package for

717	estimating above-ground biomass and its uncertainty in tropical forests. Goslee S, editor.

718	Methods Ecol Evol. 2017;8: 1163–1167. doi:10.1111/2041-210X.12753

719	59.	Martin AR, Thomas SC. A Reassessment of Carbon Content in Tropical Trees. Chave J, editor.

720	PLoS One. 2011;6: e23533. doi:10.1371/journal.pone.0023533

721	60.	Brown S. Estimating biomass and biomass change of tropical forests: A primer. Rome; 1997.

722	61.	Chave J, Réjou-Méchain M, Búrquez A, Chidumayo E, Colgan MS, Delitti WBC, et al. Improved

723	allometric models to estimate the aboveground biomass of tropical trees. Glob Chang Biol.

724	2014;20: 3177–3190. doi:10.1111/gcb.12629

725	62.	Lee YF, Chung AYC, Kitayama K. Synergy between carbon management and biodiversity

726	conservation in tropical rain forests. Proceedings on the International Workshop. 2006. p. 85.

727	63.	Ota T, Kajisa T, Mizoue N, Yoshida S, Takao G, Hirata Y, et al. Estimating aboveground carbon

728	using airborne LiDAR in Cambodian tropical seasonal forests for REDD+ implementation. J For

729	Res. 2015;20: 484–492. doi:10.1007/s10310-015-0504-3

730	64.	Ota T, Ogawa M, Shimizu K, Kajisa T, Mizoue N, Yoshida S, et al. Aboveground Biomass

731	Estimation Using Structure from Motion Approach with Aerial Photographs in a Seasonal

732	Tropical Forest. Forests. 2015;6: 3882–3898. doi:10.3390/f6113882

733	65.	Banin L, Feldpausch TR, Phillips OL, Baker TR, Lloyd J, Affum-Baffoe K, et al. What controls

734	tropical forest architecture? Testing environmental, structural and floristic drivers. Glob Ecol

735	Biogeogr. 2012;21: 1179–1190. doi:10.1111/j.1466-8238.2012.00778.x

736 66.	Siran SA, Naito R, Dharmawan IWS, Subarudi, Setyawati T. ITTO Project Technical Report PD
737 73/89 (F, M, I) Phase II: Methodology Design Document for Reducing Emissions from
738 Deforestation and Degradation of Undrained Peat Swamp Forests in Central Kalimantan,
739 Indonesia. Bogor; 2012.
740 67.	Cao L, Liu H, Fu X, Zhang Z, Shen X, Ruan H. Comparison of UAV LiDAR and Digital Aerial
741 Photogrammetry Point Clouds for Estimating Forest Structural Attributes in Subtropical

742	Planted Forests. Forests. 2019;10: 145. doi:10.3390/f10020145

743	68.	Karl JW, Yelich J V., Ellison MJ, Lauritzen D. Estimates of Willow (Salix Spp.) Canopy Volume

744	using Unmanned Aerial Systems. Rangel Ecol Manag. 2020;73: 531–537.

745	doi:10.1016/j.rama.2020.03.001

746	69.	Wich SA, Koh LP. Conservation drones: Mapping and monitoring biodiversity. Oxford: Oxford

747	University Press; 2018. doi:10.1093/oso/9780198787617.001.0001

748	70.	Giannetti F, Chirici G, Gobakken T, Næsset E, Travaglini D, Puliti S. A new approach with DTM-

749	independent metrics for forest growing stock prediction using UAV photogrammetric data.

750	Remote Sens Environ. 2018;213: 195–205. doi:10.1016/j.rse.2018.05.016

751	71.	Kachamba D, Ørka H, Gobakken T, Eid T, Mwase W. Biomass Estimation Using 3D Data from

752	Unmanned Aerial Vehicle Imagery in a Tropical Woodland. Remote Sens. 2016;8: 968.

753	doi:10.3390/rs8110968

754	72.	James MR, Robson S, D’Oleire-Oltmanns S, Niethammer U. Optimising UAV topographic

755	surveys processed with structure-from-motion: Ground control quality, quantity and bundle

756	adjustment. Geomorphology. 2017;280: 51–66. doi:10.1016/j.geomorph.2016.11.021

757	73.	Lisein J, Michez A, Claessens H, Lejeune P. Discrimination of deciduous tree species from time

758	series of unmanned aerial system imagery. PLoS One. 2015;10: e0141006.

759	doi:10.1371/journal.pone.0141006

760	74.	Roşca S, Suomalainen J, Bartholomeus H, Herold M. Comparing terrestrial laser scanning and

761	unmanned aerial vehicle structure from motion to assess top of canopy structure in tropical

762	forests. Interface Focus. 2018;8: 20170038. doi:10.1098/rsfs.2017.0038

763	75.	Zhang J, Hu J, Lian J, Fan Z, Ouyang X, Ye W. Seeing the forest from drones: Testing the

764	potential of lightweight drones as a tool for long-term forest monitoring. Biol Conserv.

765	2016;198: 60–69. doi:10.1016/j.biocon.2016.03.027

766	76.	Liu Q, Fu L, Chen Q, Wang G, Luo P, Sharma RP, et al. Analysis of the Spatial Differences in

767	Canopy Height Models from UAV LiDAR and Photogrammetry. Remote Sens. 2020;12: 2884.

768	doi:10.3390/rs12182884

769	77.	Seifert E, Seifert S, Vogt H, Drew D, van Aardt J, Kunneke A, et al. Influence of drone altitude,

770	image overlap, and optical sensor resolution on multi-view reconstruction of forest images.

771	Remote Sens. 2019;11: 1252. doi:10.3390/rs11101252

772 78.	Horning N, Fleishman E, Ersts PJ, Fogarty FA, Wohlfeil Zillig M. Mapping of land cover with
773 open-source software and ultra-high-resolution imagery acquired with unmanned aerial
774 vehicles. Remote Sens Ecol Conserv. 2020;6: 487–497. doi:10.1002/rse2.144
775 79.	Duffy JP, Cunliffe AM, DeBell L, Sandbrook C, Wich SA, Shutler JD, et al. Location, location,
776 location: considerations when using lightweight drones in challenging environments. Remote

777	Sens Ecol Conserv. 2018;4: 7–19. doi:10.1002/rse2.58

# 778 Supporting information captions

##### 779 S1 Text. Processing parameters for tree crown location estimates generated using PyCrown.

780 **S1 Table. Selected allometric equations used to generate aboveground biomass (AGB) distributions**

##### 781 from field-derived measurements.

782 AGB in kg; DBH, diameter at breast height in cm; H, tree height in m; WD, wood density in g cm^3^; W,
783 weight in kg. Forest types and underlying sample data ranges are given where available.

Figure	[Click here to access/download;Figure;Fig1.tif](https://www2.cloud.editorialmanager.com/pone/download.aspx?id=37217388&guid=8c8c3104-055a-4c36-9692-1a59da2ea013&scheme=1)

Figure	[Click here to access/download;Figure;Fig2.tif](https://www2.cloud.editorialmanager.com/pone/download.aspx?id=37217390&guid=5cb6b674-add6-47ba-ab13-c05045df3b2d&scheme=1)

Figure	[Click here to access/download;Figure;Fig3.tif](https://www2.cloud.editorialmanager.com/pone/download.aspx?id=37217392&guid=8ee37f03-a033-4b9c-b3c3-353fba9a6bca&scheme=1)

Figure	[Click here to access/download;Figure;Fig4.tif](https://www2.cloud.editorialmanager.com/pone/download.aspx?id=37217394&guid=47ad8f63-4e8f-4508-b370-b18ba2c4b28e&scheme=1)

Figure	[Click here to access/download;Figure;Fig5.tif](https://www2.cloud.editorialmanager.com/pone/download.aspx?id=37217396&guid=02a92395-fc31-41c5-8c58-f50c76b9b915&scheme=1)

Figure	[Click here to access/download;Figure;Fig6.tif](https://www2.cloud.editorialmanager.com/pone/download.aspx?id=37217399&guid=18e199a1-3722-497c-8826-90e4ff4a17e7&scheme=1)

Figure	[Click here to access/download;Figure;Fig7.tif](https://www2.cloud.editorialmanager.com/pone/download.aspx?id=37217400&guid=5294bc7c-63c8-4293-b9bc-840412e63413&scheme=1)

Supporting information table

[Click here to access/download](https://www2.cloud.editorialmanager.com/pone/download.aspx?id=37217401&guid=478a0d1b-f065-4517-8b71-e8df2061ba15&scheme=1)

[**Supporting Information** S1_Table.docx](https://www2.cloud.editorialmanager.com/pone/download.aspx?id=37217401&guid=478a0d1b-f065-4517-8b71-e8df2061ba15&scheme=1)

Supporting information text

#### [Click here to access/download](https://www2.cloud.editorialmanager.com/pone/download.aspx?id=37217402&guid=9c42f4b8-02c8-4222-bc3d-54b2f8e74c5e&scheme=1)

[**Supporting Information** S1_Text.docx](https://www2.cloud.editorialmanager.com/pone/download.aspx?id=37217402&guid=9c42f4b8-02c8-4222-bc3d-54b2f8e74c5e&scheme=1)
