# Supplementary material for: Simplifying drone-based aboveground carbon density measurements to support community forestry
Source: PLoS One. 2025 Apr 29;20(4):e0322099. doi: 10.1371/journal.pone.0322099 (PMC12040082; doi:10.1371/journal.pone.0322099)
Supplement: Attachment — Submitted filename: Response to Reviewers.docx [file pone.0322099.s005.docx]

Dear editors and reviewers,

Many thanks for considering our manuscript and sending us your comments and suggestions. Below we have listed the individual reviewer comments and the ways in which we have addressed them.

**Reviewer 1**

1. *The authors clearly presented the variability between drone-derived carbon measurements and field-based ones. I find it valuable that they pointed out the importance of choosing regionally calibrated allometric models, but it would be helpful to expand a bit more on how this influences measurement accuracy. Furthermore, developing more specific models for different types of tropical forests could indeed improve the accuracy and applicability of this approach.*
   a. **Line 354** – Added additional detail on the overestimation of ACD by generalised equations
   b. **Line 358** – Highlighted the difference in availability between individual tree and plot-aggregate equations, and how the development of more regional plot-aggregate equations would increase our method’s applicability.
2. *One aspect that stood out was the authors' consideration of the possible barriers to applying this method by community groups. While well-founded, I believe the article could benefit from suggesting practical solutions, such as training programs or partnerships that could minimize these challenges, making the method more accessible.*
   a. **Line 536** – Added a new paragraph to introduce options such as training programs and outsourcing data collection, alongside some of the potential drawbacks to these approaches that should be considered.
3. *The processing of data and associated costs were well-addressed in the text. However, it would be interesting to include more details on how to optimize these steps, especially for community groups that may not have access to high-performance equipment or software.*
   a. **Line 513** – Added nuance to the difficulties with open source programs.
   b. **Line 517** – Suggested one possible solution to lowering processing times with caveats.
   c. **Line 536** – The additional paragraph on Line 536 is perhaps also relevant for addressing this comment.
4. *In general, the writing is clear, but some sections, especially in the discussion, could be improved for better understanding. I would suggest reorganizing some parts to enhance the flow of ideas, particularly when discussing methodological limitations and how they impact the results. I have included some specific suggestions in the manuscript to simplify the text and make certain points easier to follow.*
   a. We have incorporated many of the specific suggestions that were added to the manuscript, including in-line comments (e.g., defining allometric equations; **Line 63**).
5. *One of the strengths of this manuscript is its focus on community-scale forestry projects and the role they play in both carbon sequestration and sustainable development. However, I believe the manuscript would benefit from emphasizing the broader relevance of community forestry both in the introduction and the discussion. Highlighting the unique role that community forestry plays in bridging conservation goals with local economic needs would make the study's rationale even stronger and more impactful, helping the reader understand the broader context of the study from the outset. In the discussion, the relevance of this method for community forestry could be further elaborated. Community groups often lack access to costly technology and advanced scientific methods, making low-cost, accessible approaches like the one presented in this manuscript especially valuable. This addition highlights the social and environmental impact of community forestry and reinforces the importance of making the research accessible and relevant to those who will benefit most from it.*
   a. **Lines 45 and 49** – Added additional sentences on the co-benefits of community forestry for communities and conservation alike, as well as the long-term advantages of community forestry over industrial planting/restoration projects.
   b. **Line 490** – Added a new paragraph outlining some of the secondary benefits of drones and drone data for communities who use/access them for conservation purposes. This reinforces the importance of making drones and related technologies more accessible for community groups, and the impact that scientific research and methodologies can have beyond an academic context.
   c. **Line 501** – Changed some sentences to echo the points raised in this comment.

**Reviewer 2**

1. *Please put in line 282 the variation of the mean and median of the variable "heights of the crown" (data obtained in the field), as it did in line 281 (for the data obtained with the drone).*
   a. Unfortunately we did not quite understand this comment: we have not given the variation of the mean and median for the field measurements as it was only a single distribution. The drone measurements on Line 281 have a range of values as we are summarising 6 separate distributions.
2. *The resolution of all images needs to be improved.*
   a. The images we uploaded were the correct resolution and scale according to the PLOS ONE guidelines. We notice, however, that the previews included in the document “PONE-D-24-34507_reviewer.docx” are of a much lower resolution. Hopefully this will not be the case for the final publication.

**Additional requirements**

1. *Please ensure that your manuscript meets PLOS ONE’s style requirements, including those for file naming.*
   a. This has been addressed.
2. *We note that you have indicated that there are restrictions to data sharing for this study. PLOS only allows data to be available upon request if there are legal or ethical restrictions on sharing data publicly. … b) If there are no restrictions, please upload the minimal anonymized data set necessary to replicate your study findings to a stable, public repository and provide us with the relevant URLs, DOIs, or accession numbers.*
   a. Our data has now been uploaded to the CEDA Archive, a NERC repository for earth observation data.
   b. The catalogue record link is: <https://catalogue.ceda.ac.uk/uuid/98692ec457ee431cacc4027820e46411/>
   c. The DOI (when it is issued) will be: <https://dx.doi.org/10.5285/>[98692ec457ee431cacc4027820e46411/](https://catalogue.ceda.ac.uk/uuid/98692ec457ee431cacc4027820e46411/)
3. *We note that Figure 1 and 5 in your submission contain map/satellite images which may be copyrighted. … We require you to either (1) present written permission from the copyright holder to publish these figures specifically under the CC BY 4.0 license, or (2) remove the figures from your submission.*
   a. The orthomosaic maps in Figures 1 and 5 were created by Author 1, using images that were collected by the DGFC team with a drone and used for the main analysis of this paper. We therefore do not believe that there are any copyright issues with those figures.
4. *Please review your reference list to ensure that it is complete and correct. If you have cited papers that have been retracted, please include the rationale for doing so in the manuscript text, or remove these references and replace them with relevant current references. Any changes to the reference list should be mentioned in the rebuttal letter that accompanies your revised manuscript. If you need to cite a retracted article, indicate the article’s retracted status in the References list and also include a citation and full reference for the retraction notice.*
   a. Reference list has been reviewed and the following references have been changed:
1. Tyukavina A, Baccini A, Hansen MC, Potapov P V., Stehman S V., Houghton RA, et al. Corrigendum: Aboveground carbon loss in natural and managed tropical forests from 2000 to 2012 (2015 Environ. Res. Lett. 10 074002). Environ Res Lett. 2018;13: 109501. doi:10.1088/1748-9326/aae31e
4. Olsson L, Barbosa H, Bhadwal S, Cowie A, Delusca K, Flores-Renteria D, et al. Land degradation. In: Shukla PR, Skea J, Calvo Buendia E, Masson-Delmotte V, Pörtner H-O, Roberts DC, et al., editors. Climate Change and Land: an IPCC special report on climate change, desertification, land degradation, sustainable land management, food security, and greenhouse gas fluxes in terrestrial ecosystems. Cambridge University Press; 2019. pp. 345–436. doi:10.1017/9781009157988.006
28. Cranston KA, Wong WY, Knowlton S, Bennett C, Rivadeneira S. Five psychological principles of codesigning conservation with (not for) communities. Zoo Biol. 2022;41: 409–417. doi:10.1002/zoo.21725
31. Otero V, Van De Kerchove R, Satyanarayana B, Martínez-Espinosa C, Fisol MA Bin, Ibrahim MR Bin, et al. Managing mangrove forests from the sky: Forest inventory using field data and Unmanned Aerial Vehicle (UAV) imagery in the Matang Mangrove Forest Reserve, peninsular Malaysia. For Ecol Manage. 2018;411: 35–45. doi:10.1016/j.foreco.2017.12.049
48. Zörner J, Dymond J, Shepherd J, Jolly B. PyCrown - Fast raster-based individual tree segementation for LiDAR data. Landcare Research NZ Ltd; 2018. doi:10.7931/M0SR-DN55
66. Kitayama K, editor. Co-benefits of Sustainable Forestry. Tokyo: Springer Japan; 2013. doi:10.1007/978-4-431-54141-7
b. These new references have been added:
9. Cranston KA. Building & Measuring Psychological Capacity for Biodiversity Conservation. Antioch University, New England. 2016. Available: https://aura.antioch.edu/etds/293
12. Duguma L, Minang P, Aynekulu E, Carsan S, Nzyoka J, Bah A, et al. From Tree Planting to Tree Growing: Rethinking Ecosystem Restoration Through Trees. Nairobi; 2020. Report No.: 304. doi:10.5716/WP20001.PDF
13. Fox H, Cundill G. Towards Increased Community-Engaged Ecological Restoration: A Review of Current Practice and Future Directions. Ecol Restor. 2018;36: 208–218. doi:10.3368/er.36.3.208
14. Kodikara KAS, Mukherjee N, Jayatissa LP, Dahdouh-Guebas F, Koedam N. Have mangrove restoration projects worked? An in-depth study in Sri Lanka. Restor Ecol. 2017;25: 705–716. doi:10.1111/rec.12492
15. Martin MP, Woodbury DJ, Doroski DA, Nagele E, Storace M, Cook-Patton SC, et al. People plant trees for utility more often than for biodiversity or carbon. Biol Conserv. 2021;261: 109224. doi:10.1016/j.biocon.2021.109224
16. Coleman EA, Schultz B, Ramprasad V, Fischer H, Rana P, Filippi AM, et al. Limited effects of tree planting on forest canopy cover and rural livelihoods in Northern India. Nat Sustain. 2021;4: 997–1004. doi:10.1038/s41893-021-00761-z
17. Lewis SL, Wheeler CE, Mitchard ETA, Koch A. Restoring natural forests is the best way to remove atmospheric carbon. Nature. 2019;568: 25–28. doi:10.1038/d41586-019-01026-8
70. Hao H, Li W, Zhao X, Chang Q, Zhao P. Estimating the Aboveground Carbon Density of Coniferous Forests by Combining Airborne LiDAR and Allometry Models at Plot Level. Front Plant Sci. 2019;10: 917. doi:10.3389/fpls.2019.00917
71. Cushman KC, Burley JT, Imbach B, Saatchi SS, Silva CE, Vargas O, et al. Impact of a tropical forest blowdown on aboveground carbon balance. Sci Rep. 2021;11: 11279. doi:10.1038/s41598-021-90576-x
72. Nunes M, Ewers R, Turner E, Coomes D. Mapping Aboveground Carbon in Oil Palm Plantations Using LiDAR: A Comparison of Tree-Centric versus Area-Based Approaches. Remote Sens. 2017;9: 816. doi:10.3390/rs9080816
87. Newport B. Going vertical: Exploring the technical opportunities and socio-political dynamics of drones in forest conservation. University of Bristol. 2024. Available: https://research-information.bris.ac.uk/en/studentTheses/going-vertical
88. Radjawali I, Pye O, Flitner M. Recognition through reconnaissance? Using drones for counter-mapping in Indonesia. J Peasant Stud. 2017;44: 817–833. doi:10.1080/03066150.2016.1264937
90. Gülci S. The determination of some stand parameters using SfM-based spatial 3D point cloud in forestry studies: an analysis of data production in pure coniferous young forest stands. Environ Monit Assess. 2019;191: 495. doi:10.1007/s10661-019-7628-4
91. Sandbrook C, Luque-Lora R, Adams WM. Human Bycatch: Conservation Surveillance and the Social Implications of Camera Traps. Conserv Soc. 2018;16: 493–504. doi:10.4103/cs.cs_17_165
92. Millner N, Newport B, Sandbrook C, Simlai T. Between monitoring and surveillance: Geographies of emerging drone technologies in contemporary conservation. Prog Environ Geogr. 2024;3: 17–39. doi:10.1177/27539687241229739
93. Cummings AR, Cummings GR, Hamer E, Moses P, Norman Z, Captain V, et al. Developing a UAV-Based Monitoring Program with Indigenous Peoples. J Unmanned Veh Syst. 2017; 115–125. doi:10.1139/juvs-2016-0022
94. Sauls LA, Paneque-Gálvez J, Amador-Jiménez M, Vargas-Ramírez N, Laumonier Y. Drones, communities and nature: pitfalls and possibilities for conservation and territorial rights. Glob Soc Challenges J. 2023;2: 24–46. doi:10.1332/AJHA9183
95. Vargas-Ramírez N, Paneque-Gálvez J. The Global Emergence of Community Drones (2012–2017). Drones. 2019;3: 76. doi:10.3390/drones3040076

We would like to thank both reviewers for taking the time to engage with our manuscript and for supporting what we hope is an improved final article.
